# Stepwise management of hepatocellular carcinoma associated with Abernethy syndrome

**DOI:** 10.1002/ccr3.1384

**Published:** 2018-03-30

**Authors:** Niki Christou, Nabil Dib, Etienne Chuffart, Abdelkader Taibi, Sylvaine Durand‐Fontanier, Denis Valleix, Muriel Mathonnet

**Affiliations:** ^1^ Department of Digestive and Endocrine Surgery University Hospital of Limoges 2 avenue Martin Luther King 87042 Limoges Cedex France

**Keywords:** Abernethy syndrome, congenital absence of the portal vein, hepatocellular carcinoma, portocaval shunt

## Abstract

Patients with congenital agenesis of the portal vein may develop hepatocellular tumors due to enhanced arterial blood flow. These tumors may be benign (FNH, adenomas) or malignant (hepatoblastoma, HCC). Liver resection can be proposed, and preoperative arterial embolization may decrease blood loss during surgery. Liver transplantation with PV reconstruction is also an option.

## Introduction

Abernethy syndrome is a congenital disease characterized by the total or partial agenesis of the portal vein, creating a portosystemic shunt in the absence of portal hypertension or liver disease. This congenital absence of the portal vein (CAPV) is discovered during childhood in 80% of cases, but may also be diagnosed in adults, in a context of complications.

This syndrome is rare and has been reported only about a hundred times since its description by John Abernethy in 1793 1. The number of cases of Abernethy syndrome has increased in recent years, due to advances in imaging techniques 2.

In normal physiological conditions, the portal vein is formed by the superior mesenteric vein and the spleen mesaraic trunk, which is itself made up of the inferior mesenteric vein and the splenic vein. The blood enters the liver for purification and the hepatic veins then penetrate into the inferior vena cava. In Abernethy syndrome, the liver is bypassed and the unpurified blood passes directly into the systemic circulation via the inferior vena cava.

Two types of Abernethy syndrome have been described: type I, corresponding to a congenital absence of the portal vein, and type II, in which blood supply to the portal vein is partly preserved 3. Type I Abernethy syndrome can be split into two subtypes, 1a and 1b, depending on the configuration of the junction between the spleen mesaraic trunk and the vena cava:

1a: the spleen mesaraic trunk and the superior mesenteric vein flow directly into the inferior vena cava
1b: the spleen mesaraic trunk and the superior mesenteric vein join to form a trunk vein that then plunges into the inferior vena cava

In type II Abernethy syndrome, a portal vein is present, but it is hypoplasic due to a predominant shunt between the portal vein and the inferior vena cava.

Type I predominates in female subjects (61–78%) and includes multiple malformations, some of which may be lethal 4. Type II displays a slight male predominance (50–70%) 4. Both types are commonly complicated by hepatic encephalopathy in adults, but other complications, such as liver nodules or hepatocellular carcinoma (HCC), may also arise and may even lead to discovery of the syndrome 5.

Liver transplantation appears to be the most effective treatment in children and young adults 4, 6, 7. In adults, CAPV is often diagnosed following the occurrence of a large hepatic tumor, precluding liver transplantation. We describe here the management of a large tumor associated with Abernethy syndrome.

## Steps in the Management of Abernethy Syndrome

### Step 1: Preoperative work‐up: imaging analysis

**Figure 1:**
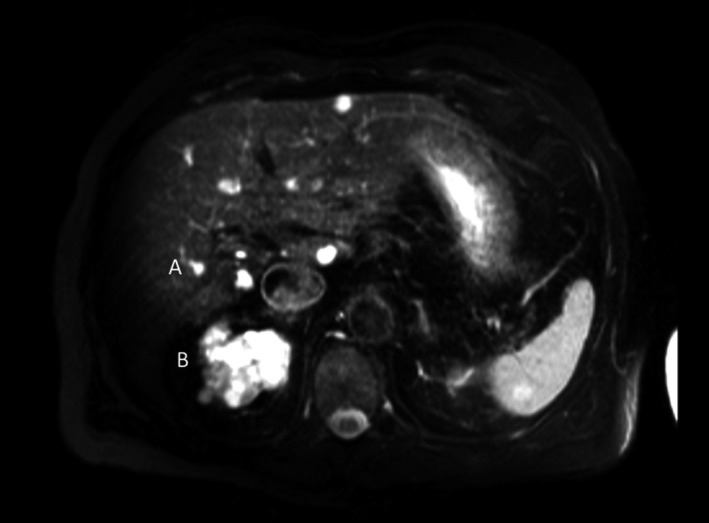
Two years before the development of HCC, T2‐weighted MRI revealed the presence of liver cysts (A) and renal polycystic kidneys.

Mr. D., aged 72, had been followed for more than 15 years by hepatologists and nephrologists, for hepatorenal polycystic kidney (HRPK) disease associated with microbiliary hamartomas (Fig. 1). He also had high blood pressure and suffered a myocardial infarction in 2001. His HRPK was stable in both the liver and kidneys, and ultrasound scans or magnetic resonance imaging (MRI) was performed regularly, at two‐year intervals. No malformations were noted until 2012.

**Figure 2:**
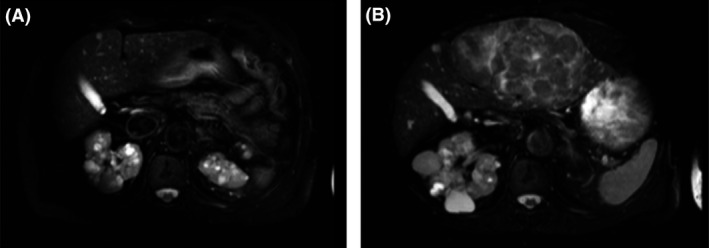
Findings of the T2‐weighted MRI. (A) Two years previously, liver MRI was normal. (B) The whole left hepatic lobe was deformed due to a heterogeneous tumor. No lymph node enlargement or ascites were observed.

In April 2013, the patient was in poor general condition and had lost about 5 kg in weight without anorexia. Ultrasound imaging of the liver revealed a single tumor in the left lobe of the liver, with a major axis of 15 cm in length and characteristics suggestive of hepatocellular carcinoma (HCC) (Fig. 2). These findings were confirmed by MRI.

**Figure 3:**
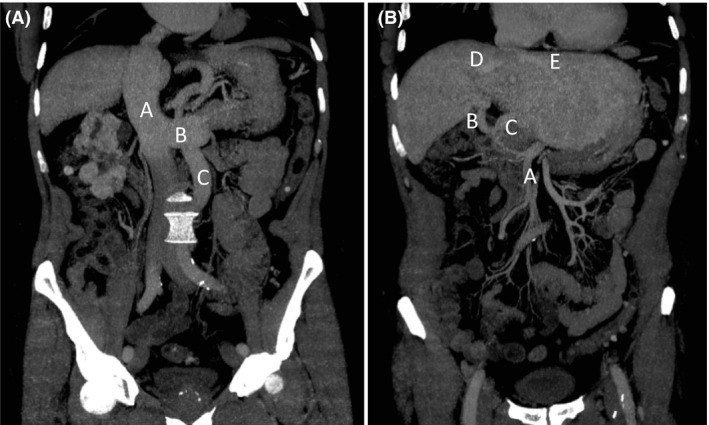
The coronal section of the CT scan showed (A) a type 1a Abernethy malformation, with complete agenesis of the portal vein, and (B) the arterialisation of the liver before embolization and the absence of the middle hepatic vein. (A) A: vena cava; B: left renal vein; C: inferior mesenteric vein. (B) A: superior mesenteric artery; B: right hepatic artery; C: left hepatic artery; D: right hepatic vein; E: left hepatic vein.

The results of laboratory tests, for liver transaminases, CA 19.9, AFP, and CEA, were normal. Serological tests for hydatid cysts, echinococcosis, and amebiasis were negative. Oeso‐gastroduodenal endoscopy showed no signs of portal hypertension. CT scan confirmed the diagnosis of HCC and revealed two malformations. The first was a type Ia Abernethy malformation, with complete agenesis of the portal vein, deviation of all portal flow in the left renal vein, and liver arterialisation. The second was the absence of the middle hepatic vein (HV). There were only two HVs, one on the right and the other on the left (Fig. 3). The HCC had developed in the left lobe of the liver. CT scan showed that resection with preservation of the vessels in the right lobe of the liver was possible. Resection of the left lobe was therefore proposed.

### Step 2: Preoperative preparation

Three days before surgery, preoperative embolization of the left hepatic artery was performed, and biochemical data were collected for the liver.

### Step 3: Preoperative management

An ultrasound scan of the liver was performed during surgery, to localize the two hepatic veins (left and right HVs) and their confluence and the junction with the inferior vena cava. The left HV passed through the tumor, and the right HV was located close to the tumor. The gastrohepatic ligament was opened and the hepatic pedicle was carefully explored: there was no portal trunk, the right liver artery was recovered and the left was embolized. A left hepatectomy was performed by an anterior approach, and biliary stasis was checked by cholangiography after the cholecystectomy. The hepatic section was in contact with the right hepatic vein. The transfusion of only two units of packed cells was required during surgery. The patient did not develop hepatocellular insufficiency. On D2, ALAT levels had almost returned to normal (114 U/mL), bilirubin concentration was 29 μmol/L and the patient had a prothrombin ratio of 69%. Laboratory test results returned to normal values by D10. The patient was discharged from the digestive medicine unit 10 days after surgery.

**Figure 4:**
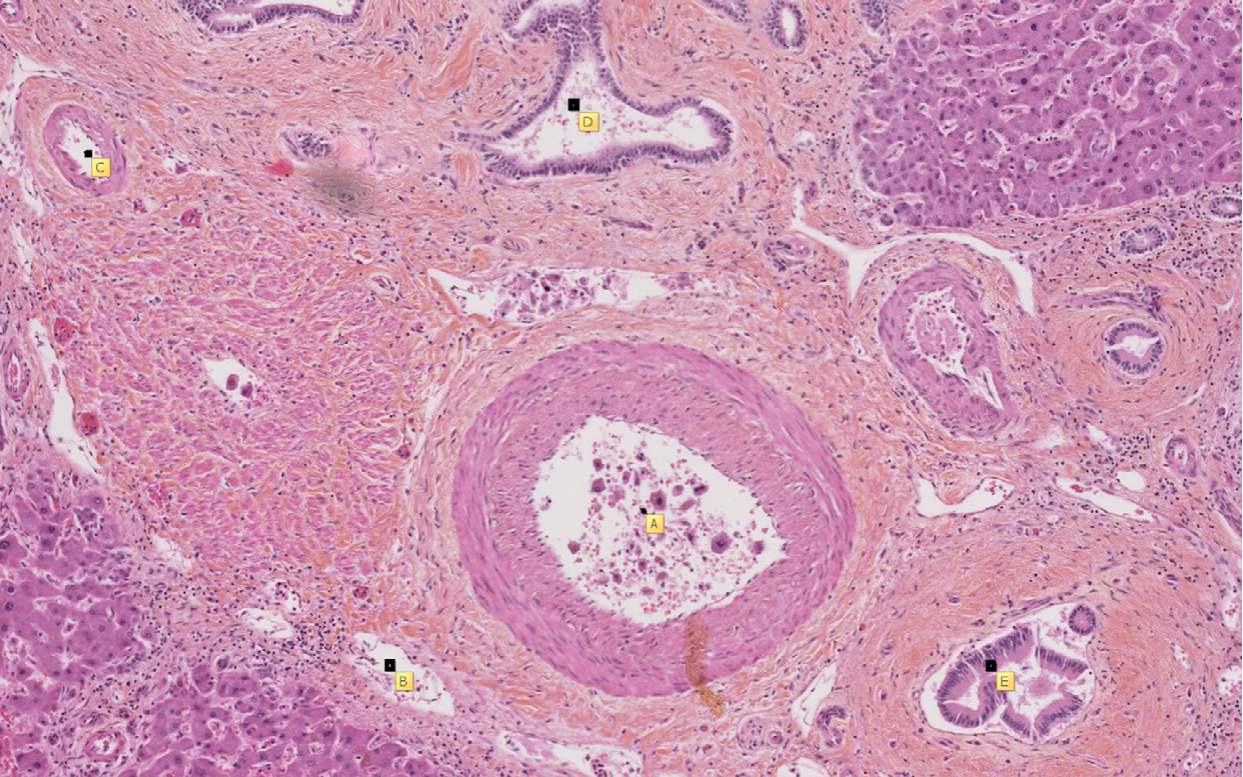
Hematoxylin‐eosin‐saffron staining, magnification ×50. A voluminous portal space fused to microbiliary hamartomas. (A) Branch of the hepatic artery; (B) a dysmorphic vein; (C) duplicated arteries from the “1” artery; (D) biliary microhamartomas; (E) a bile duct.

The final pathology evaluation reported a well‐differentiated HCC with invasion of the channel section. Two types of lesions were also observed: biliary microhamartomas and arterial branch duplication, with an absence of venous branches in the portal spaces, and fibrosis (Fig. 4).

## Discussion

In adults, Abernethy syndrome type I, or CAPV, is often associated with large liver tumors, but it may also be associated with other, possibly lethal malformations (Table 1) 4.

**Table 1:** Anomalies associated with congenital portosystemic shunts, and their potential lethality

Organ	Type of malformation	Fatal issue
Heart	Inter‐auricular communication, permeable ductus, inter ventricular communication, transposition of large vessels, situs inversus, aortic coarctation, lethal congenital cardiomyopathy	Possibly
Spleen	Polysplenia	No
Kidney	Cystic renal dysplasia, bilateral ureteropelvic obstruction	No
Bone	Vertebral abnormality in the type of hemivertebra, short appearance of the 5th fingers and toes	No
Liver	Hepatic tumors (hepatocellular carcinomas hepatoblastomas), abnormalities of the bile ducts, anomaly of the hepatic balance to the type of cytolysis and cholestasis	Possibly
Miscellaneous	Non functioning pancreatic tumor, ulcerative colitis	Possibly

It may also have diverse complications, which may lead to detection of the syndrome:

1. Encephalopathy with hyperammonemia due to an absence of blood detoxification resulting from the portosystemic shunt;
2. Brain abscess;
3. Hepatopulmonary syndrome, which causes chronic hypoxia with clinical signs, such as cyanosis, dyspnoea, and nail clubbing.

The vein usually arises by selective involution of the peri‐intestinal vitelline venous loop during early embryogenesis. Excessive involution of this loop can cause congenital agenesis of the portal vein, with the development of a complete or partial mesocaval shunt, as reported here 3, 4.

Moreover, about half the individuals with congenital agenesis of the portal vein also have benign or malignant liver neoplasms 5, mostly focal nodular hyperplasia 8, or more rarely an adenoma 9, nodular regenerative hyperplasia 5, or hepatocellular carcinoma, as in our case.

The high frequency of liver disease associated with congenital agenesis of the portal vein strongly suggests a relationship between the systemic deviation of portal flow and the development, hepatocellular function, and regenerative capacity of the liver parenchyma 10. Humans display an arterioportal equilibrium referred to as the “buffer response,” corresponding to an increase in arterial flow when portal flow decreases, but no increase in portal flow when arterial flow decreases. The nodules are histologically variable, and their development seems to depend on arterial blood supply 4. The HCC of our case seems to be related to a high degree of arterialisation. Moreover, the biliary hamartomas associated with the HCC are typical abnormalities of the bile ducts often associated with Abernethy syndrome. Hepatorenal polycystic disease is the most common hereditary disease affecting the kidneys. This patient was followed for more than 15 years by hepatologists and nephrologists, but the malformations of the vessels in the liver remained undetected before the onset of HCC.

Imaging revealed a direct deviation of portal venous blood in our case, with the upper and lower mesenteric veins and the splenic vein emptying directly into the left renal vein, and a high level of liver arterialisation. No signs of portal hypertension were observed, but high levels of blood flow through the hypertrophic hepatic artery, associated with bile duct proliferation, were observed on pathology examination 11.

In our case, the liver was resected after chemical embolization, due to the large size of the tumor. The preoperative embolization of the left artery significantly decreased blood loss. We felt that mobilization of the tumor before liver resection was too dangerous, and an anterior approach therefore remained the only viable option.

Portosystemic caval shunts can lead to abnormal liver development, function, and regeneration due to the absence of portal hepatotrophic factor, resulting in the development of hepatic tumors. These tumors are generally considered to be benign parenchymatous lesions, but with the potential for malignant conversion 12.

In young patients with immature intrahepatic portal veins, as in our case, liver transplantation (LT) may be required in young patients. Surgical reconstruction of the portal structures of the native liver could be proposed, but this method has yet to be clearly established.

Two methods can be used to reconstruct the portal vein during LT LT for CAPV. In the first, the shunt is anastomosed end‐to‐end to the portal vein in the graft 8.

The other technique involves the anastomosis, end‐to‐side, of a venous interposition graft to the shunt via a partial side clamp, with the venous interposition graft anastomosed end‐to‐end to the portal vein in the graft 13.

**Figure 5:**
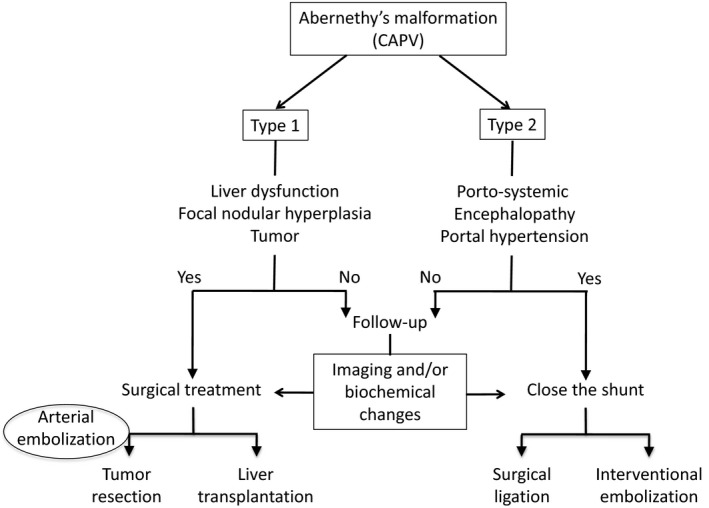
Decision algorithm for the management of patients with complete agenesis of the portal vein.

However, it is important to check the feasibility of LT in cases of type I portocaval shunt, in which the portal vein is immature, as in our case. Indeed, it is necessary to analyze tumor size, histology, and residual liver volume. Embolisation significantly decreases the amount of blood lost and facilitates the resection. As suggested by Hua, we propose the use of a decision algorithm for the management of patients with CAPV (Fig. 5).

## Conclusion

In conclusion, we describe a case of congenital agenesis of the portal vein diagnosed in a 72‐year‐old man, a much greater age at diagnosis than in previous studies, which have focused on the frequent association of liver damage with this congenital malformation. This vein agenesis seemed to be primarily a consequence of hepatic hyper arterialisation linked to the deprivation of portal blood flow. Surgery is the treatment of choice for this disease when associated with the formation of a liver tumor, as in this case. The decision as to whether to perform LT or liver resection‐chemoembolisation is based on the same criteria as for HCC.

## Conflict of Interest

None declared.

## Authorship

NC and ND: analyzed the data and wrote this clinical case. EC, AT, SDF, and DV: contributed to interpret the images and revised the clinical case. MM: designed, revised, and approved the final version of the clinical case.
